# Dolphin-WET—Development of a Welfare Evaluation Tool for Bottlenose Dolphins (*Tursiops truncatus*) under Human Care

**DOI:** 10.3390/ani14050701

**Published:** 2024-02-23

**Authors:** Katrin Baumgartner, Tim Hüttner, Isabella L. K. Clegg, Manuel Garcia Hartmann, Daniel Garcia-Párraga, Xavier Manteca, Birgitta Mercera, Tania Monreal-Pawlowsky, Cristina Pilenga, Kerstin Ternes, Oriol Tallo-Parra, Ruta Vaicekauskaite, Lorenzo von Fersen, Lisa Yon, Fabienne Delfour

**Affiliations:** 1 Behavioral Ecology and Conservation Lab, Nuremberg Zoo, 90480 Nuremberg, Germany; 2 Animal Welfare Expertise, Winchester SO22 6QU, UK; 3 MarLab, 06250 Mougins, France; 4 Fundación Oceanográfic de la Comunitat Valenciana, 46013 Valencia, Spain; 5 Animal Welfare Education Centre (AWEC), Veterinary Faculty, Universitat Autònoma de Barcelona, 08193 Barcelona, Spain; 6 Parc Astérix, 60128 Plailly, France; 7 International Zoo Veterinary Group, Keighley BD21 4NQ, UK; 8 Zoomarine Italia, 00071 Rome, Italy; 9 Zoo Duisburg, 47058 Duisburg, Germany; ternes@zoo-duisburg.de; 10 Fox Consulting, 67500 Haguenau, France; ruta@fox-zooconsulting.com; 11 Faculty of Medical & Health Sciences, School of Veterinary Medicine and Science, The University of Nottingham, Sutton Bonington, Leicestershire LE12 5RD, UK; 12 Ecole Nationale Vétérinaire de Toulouse, 31076 Toulouse, France; 13 Animaux et Compagnies, 31500 Toulouse, France

**Keywords:** cetacean welfare, welfare evaluation, welfare indicator, marine mammals, zoo animals

## Abstract

**Simple Summary:**

The welfare committee of the European Association for Aquatic Mammals (EAAM) set up a group of experts on welfare science, cetacean biology, and zoo animal medicine across Europe to develop a comprehensive tool to evaluate the welfare of bottlenose dolphins (*Tursiops truncatus*) under human care named Dolphin-WET. The tool encompasses 49 indicators (i.e., 37 animal-based and 12 resource-based indicators) inspired by Mellor’s Five Domains Model and the Welfare Quality^®^. The Dolphin-WET is a species-specific and individual-based welfare assessment tool that provides a holistic approach to evaluating bottlenose dolphins’ welfare.

**Abstract:**

Ensuring high standards of animal welfare is not only an ethical duty for zoos and aquariums, but it is also essential to achieve their conservation, education, and research goals. While for some species, animal welfare assessment frameworks are already in place, little has been done for marine animals under human care. Responding to this demand, the welfare committee of the European Association for Aquatic Mammals (EAAM) set up a group of experts on welfare science, cetacean biology, and zoo animal medicine across Europe. Their objective was to develop a comprehensive tool to evaluate the welfare of bottlenose dolphins (*Tursiops truncatus*), named Dolphin-WET. The tool encompasses 49 indicators that were either validated through peer review or management-based expertise. The first of its kind, the Dolphin-WET is a species-specific welfare assessment tool that provides a holistic approach to evaluating dolphin welfare. Inspired by Mellor’s Five Domains Model and the Welfare Quality^®^, its hierarchical structure allows for detailed assessments from overall welfare down to specific indicators. Through combining 37 animal-based and 12 resource-based indicators that are evaluated based on a two- or three-level scoring, the protocol offers a detailed evaluation of individual dolphins. This approach allows for regular internal monitoring and targeted welfare management, enabling caretakers to address specific welfare concerns effectively.

## 1. Introduction

Responsible zoos and aquariums claim guardianship for species conservation, research, and education. The basic prerequisite, however, is to work towards and to provide high standards of animal welfare for the animals under their care. Through combining scientific and management-based expertise (MBE), modern zoos work towards developing methods for assessing and continuously improving the welfare of animals. The term “welfare” encompasses both physical and mental health, considering that the animals’ opportunities for choice and control relate to both the physical and social resources available in their habitats and requires an understanding of how the animals perceive and cope with a situation. “Welfare” is individual because it is affected by an animal’s age, class, sex, life history, and health status, among other factors. Due to the complexity of animal welfare and its assessment, numerous parameters must be evaluated to provide a holistic appraisal of an animal’s welfare state [1,2,3]. Early zoo animal welfare assessments took inspiration from the Farm Animal Welfare Council [4], updates of the five freedoms from the Welfare Quality^®^ project—a hierarchical and widely used tool used to measure the welfare of farm animals—and more recently from the Five Domains Model [5,6]. Contemporary welfare assessments aim to capture physical and mental welfare states and encompass negative and positive welfare indicators in these five domains. Thus, there is a need to develop frameworks that incorporate both animal- and resource-based indicators to generate a complete assessment on the welfare state of an animal [7,8]. Animal-based indicators include measurements that focus on the actual welfare of an animal, considering its behaviour, mental state, health, and nutrition [5]. Resource-based indicators address questions regarding housing, management, or other essential provisions necessary for an animal’s welfare. Behavioural observations have been one of the most commonly used methods to gather animal-based measures, particularly for welfare assessments in zoo animals [9]. Behavioural studies provide information on the occurrence, duration, and frequency of behaviours that may indicate a positive welfare state (e.g., play behaviour), or other positive social interactions (e.g., affiliative behaviours); they can also provide information on the frequency of stereotypic or other abnormal behaviours, which may indicate a welfare concern. Recently, the evaluation of the mental state of an animal (e.g., emotions and cognitive biases) has also become a key focus of animal welfare research, as it is part of the Five Domains approach.

Several welfare assessment protocols are already in place for different zoo species, including polar bears [10], elephants [2], macaques [11], Dorcas gazelles [12], and giraffes [13], among others. Tallo-Parra et al. [14] provide an overview of existing zoo animal welfare assessments and discuss the most widely accepted animal-based indicators of animal welfare, highlighting the areas that require further research.

There have been several recent projects to develop and validate welfare assessment tools in terrestrial mammals. In dolphins and other aquatic mammals, however, although there has been quite a bit of work to this day, no consensus could be established on the best method and indicators to evaluate welfare in species such as the bottlenose dolphin (*Tursiops truncatus*). There have been several studies that investigated how different factors (e.g., environmental enrichment) impact the welfare of bottlenose dolphins, harbor seals (*Phoca vitulina*), California sea lions (*Zalophus californianus*), or beluga whales (*Delphinapterus leucas*) [15,16,17,18,19,20]. In a collaborative approach led by Alliance of Marine Mammal Parks and Aquariums (AMMPA) and Association of Zoos and Aquariums (AZA) members, the “Cetacean Welfare Study” (CWS) project, a multi-facility investigation, was initiated. It has involved several studies identifying potential welfare indicators and investigating the impact of various factors (e.g., housing, management, training, and enrichment procedures) on the welfare of dolphins under human care [21,22,23,24,25]. For example, dolphins engaged in more social interactions and exhibited high group activity rates when new enrichment devices were regularly provided [26]. Recent studies have pointed out several other promising potential welfare indicators: the behavioural diversity index, which refers to the frequency and variability of species-specific behaviours displayed by an individual, suggests that high behavioural diversity may indicate a positive welfare state [3,27]; predictable training schedules [24] linked to anticipatory behaviour (another potential welfare indicator) [28,29]; the dolphins’ willingness to participate in training activities [15,30,31]; post-conflict behaviours that structure dolphins’ social networks [32]; and swimming features as potential indicators of the dolphins’ emotional states [33]. Despite all these studies, to date, there is still no individual-based welfare assessment tool specifically developed for bottlenose dolphins under human care that includes a wide range of animal- and resource-related indicators.

However, in scientifically managed modern zoos, the assessment of dolphin welfare has been a major concern for years. Starting in the early 1990s, methods for evaluating aquatic animal welfare became more standardized via monitoring animal behaviour, social interactions, breeding frequency, and blood cortisol levels in different groups of bottlenose dolphins under human care [34]. In 2012, the European Association for Aquatic Mammals (EAAM) established its Welfare Committee to emphasize the importance of welfare for marine animal husbandry. The C-Well^®^ [35] protocol represented the first comprehensive tool to systematically measure the welfare of bottlenose dolphins under human care and included 36 species-specific indicators; this tool has served as an important starting point for developing this methodology. In 2016, a workshop was held at Nuremberg Zoo in Germany, hosted by the European Association of Zoos and Aquaria (EAZA), the Verband der Zoologischen Gaerten (VdZ) Alliance, the World Association of Zoos and Aquariums (WAZA), European Parliament (EP) Intergroup Climate Change, and EAAM, in cooperation with the Nuremberg Zoo. The aim of the workshop was to identify objective welfare indicators in marine mammals, especially in cetaceans under human care [36] and resulted in an animal welfare decision tree (AWDT) that includes a four-step evaluation of marine mammal welfare for external audits [37]. Inspired by the C-Well^®^ protocol [35], the board of the EAAM commissioned the Welfare Committee to create the Dolphin Welfare Evaluation Tool (Dolphin-WET). Using some measures already included in the C-Well^®^ but focusing on proposing new criteria, the main goal in developing the Dolphin-WET was to create a tool that would provide an internal objective evaluation of the welfare of individual dolphins over time, unlike previous attempts that were designed for external assessments (C-Well^®^ and AWDT).

## 2. Dolphin-WET Development

Indicators and protocols to evaluate animal welfare are highly dependent on the definition of welfare used, as well as the underlying theoretical approach. In the present study, we have defined animal welfare as: “An ongoing positive physical and mental state resulting from the satisfaction of the animal’s behavioural and physiological needs and expectations. This state varies according to the perception of the situation by the animal” [38]. This explicative and operational definition relates to the actual Five Domains Model of Mellor et al. (2020). Hence, our theoretical approach is more mental than naturalist or adaptative [39,40], and allows us to consider the animals’ mental states (e.g., emotions).

The main objective of the Dolphin-WET is to develop a rigorous and scientifically based welfare tool that uses information and data from peer-reviewed journals and experts’ (i.e., biologists, ethologists, and veterinarians) knowledge to objectively measure the welfare of bottlenose dolphins under human care. The tool was designed to be carried out routinely by the dolphins’ caretakers and to be used as an internal tool for monitoring and improving the welfare of individuals. This ensures that both adequate and inadequate welfare states are identified at the individual level and that actions can be taken accordingly. Finally, to be efficient, an evaluation should be rapid, minimally invasive, and not require special equipment or specific animal training [2].

**Figure 1:**
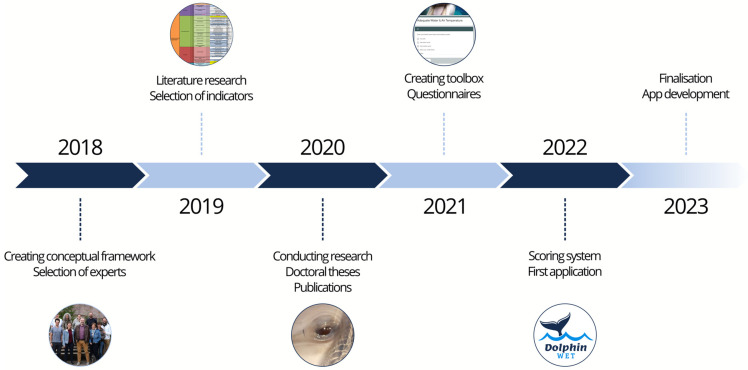
Dolphin Welfare Evaluation Tool (Dolphin-WET) development timeline. The initial framework was created by the European Association for Aquatic Mammals (EAAM) Welfare Committee and a selected group of experts in 2018. Literature research, research projects, and other activities led to the creation of a proposed framework in 2022 that was then applied in different dolphinaria for the first time. Using the information from the first application, the tool was finalised in 2023.

The development of the tool started in 2018 with the creation of a conceptual framework, followed by the selection of the principles, criteria, sub-criteria, and indicators in 2019. Continuing with several research projects and doctoral theses, including questionnaires, to the creation of a toolbox and a scoring evaluation system, the Dolphin-WET took almost six years to complete (Figure 1).

### 2.1. Working Group Composition

Chaired by the EAAM Welfare Committee, the working group in charge of the conceptualisation, development, and general review included committee members as well as external experts from a wide range of welfare-relevant fields such as veterinarians, behavioural biologists, welfare experts, trainers, and ex situ researchers from different facilities or universities. The aim was to create a heterogenous group of experts on welfare science, cetacean biology and behaviour, and veterinary medicine. Thus, both practical experience in animal handling and veterinary care and scientific knowledge were combined with the current state of animal welfare research.

### 2.2. Scientific Literature Research

An initial important phase was to review the scientific literature on the topic. This included all relevant publications on the evaluation of welfare, but also on behaviour, nutrition, husbandry, health, and mental state, not only on dolphins but also on other species for which such literature was available. In this first review process, more than 200 scientific publications were included. To obtain a better overview of the existing literature and to include relevant welfare components, the papers were then assigned to the different principles considered in the tool.

### 2.3. Structure

**Figure 2:**
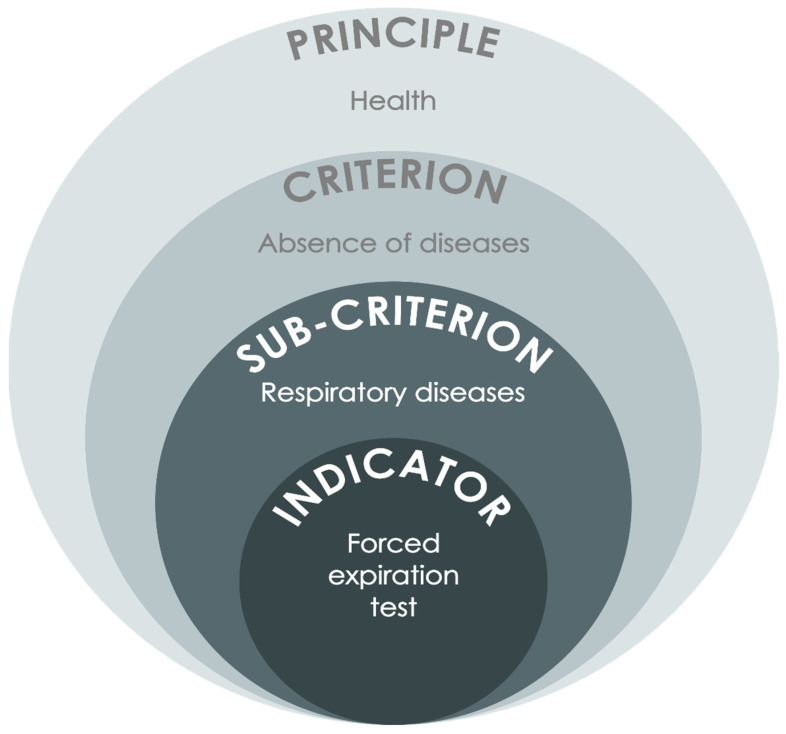
Structure of the Dolphin Welfare Evaluation Tool (Dolphin-WET) using the example of the indicator *Forced expiration test* within the principal *Health* to evaluate the condition of the respiratory tract.

The conceptual structure of the tool was inspired by animal welfare assessment grids developed for other species (e.g., the Welfare Quality^®^ Assessment Protocol). We selected the main principles for the Dolphin-WET by following the Five Domains Model [5,41]: nutrition, environment, health, behaviour, and mental state. Each principle includes several welfare criteria and sub-criteria. Each sub-criterion is measured via different indicators (Figure 2). Importantly, the present protocol prioritises animal-based indicators, and it does not allow compensation between principles or between criteria or sub-criteria from the same principle. During the tool development process, 5 principles, 18 criteria, and 38 sub-criteria were defined. The list of indicators was reduced step-by-step from over 60 to the 50 most relevant ones (12 resource-based indicators and 38 animal-based indicators; see Table 1), with representation from all welfare dimensions.

### 2.4. Selection of the Principles, Criteria, Sub-Criteria, and Indicators

To assess animal welfare, it is mandatory to identify reliable indicators that reflect how the animals are coping with the present situation. An indicator must describe the state, level, or intensity of a factor and must include the following principles: validity, reliability, and feasibility [42]. Validity means that the indicator must be meaningful for an animal and must measure what it is supposed to; reliability means that consistent results are produced when different observers use the indicator; and, finally, feasibility means it is easy to use [43].

### 2.5. Validation of the Indicators

The selected indicators must be scientifically validated whenever possible or rely on MBE [44], which refers to a source of knowledge based on years of experience in caring for and managing animals under human care. This practical knowledge (i.e., expertise) can be considered equivalent to science-based information [45].

Welfare indicators have been validated by previous peer-reviewed studies based on existing MBE, including the *EAAM Standards and Guidelines for the Management of Aquatic Mammals Under Human Care (Version March 2019)* [46] (from now on, only referred to as EAAM Standards and Guidelines) compiling experts’ criteria (e.g., safe environment, adequate water pH, or the presence of shadow areas), by research studies conducted by members of the Dolphin-WET group, or by using self-developed online questionnaires sent out to all EAAM members that addressed specific aspects (e.g., adequate diet and temperature).

#### 2.5.1. Surveys

The EAAM Welfare Committee specifically designed questionnaires for the Dolphin-WET to better understand the importance of some parameters (e.g., food variety, water, and air temperature) for the dolphins’ welfare. This approach provided a way to rapidly collect experienced dolphin caretakers’ feedback (i.e., MBE) and to incorporate the results of those surveys as potential (and need to be further scientifically validated) indicators into the Dolphin-WET.

These questionnaires were sent to all EAAM member institutions with the request to be completed by dolphin caretakers with at least 10 years of experience in handling cetaceans. A total of 14 questionnaires from 10 different institutions were returned on the topic of “adequate fish variety”, and 15 questionnaires from 9 institutions were returned on the topic of “adequate air and water temperature”. Of course, we did not assume that implemented management and husbandry procedures are automatically appropriate in terms of welfare. However, we think that decades of experience and routinely collected data (i.e., clinics, reports, etc.) from the zoo community have provided sufficient evidence that the living conditions provided (e.g., water temperature) are indicative of an adequate welfare state. Thus, for now and whilst waiting for scientific validation, we have included these data in the Dolphin-WET.

##### Survey Results for “Adequate Food Variety”

In the wild, bottlenose dolphins are opportunistic feeders, preying on a wide range of fishes and molluscs depending on seasonality, habitat, and sex [47,48]. Consequently, for husbandry, it is important to provide a variety of food. Therefore, in the questionnaires, the caretakers were asked to provide information on their selection of food species (e.g., preferences, composition, and variety) and supplementation, and if those factors showed any correlation with the observed changes in the dolphins’ behaviour or health state.

The results of the survey showed that dolphin caretakers think it is important for the dolphins to feed on at least 3–5 different species to guarantee a rich nutritional profile and to provide food rations during trainings, but also via enrichment (e.g., feeder puzzles), or during a non-training interaction with animal keepers. Moreover, each animal’s preferences or dislikes for specific species as well as dietary concerns are considered. The quantity of fish fed during training sessions should vary between sessions to add variety in reinforcement (e.g., based on session type).

##### Survey Results for “Adequate Air and Water Temperature”

We wanted to investigate whether EAAM dolphin caretakers notice differences in the animals’ overall behaviour when considering the temperature ranges included in the EAAM Standards and Guidelines (14–30 °C).

In all but one facility (*n* = 9), the food intake for the dolphins is adjusted according to the water temperature. In terms of animal welfare, 9 out of 15 dolphin caretakers observed changes in the dolphins’ behaviour according to high or low water or air temperatures. Moreover, very high or low air or water temperatures (i.e., at either end of the EAAM temperature range) appear to affect the dolphins’ willingness to participate in training sessions, their general activity and appetite, and the occurrence of social and sexual behaviour. Out of the 15 responses, 5 reported a link between increased water temperatures and the observed behavioural changes (e.g., reduced motivation and appetite with higher water temperatures), and 5 reported that water and air temperatures impacted the dolphins’ health. Considering the dolphins’ health status, dolphin caretakers reported a decline in overall health when either water temperatures (*n* = 6), air temperatures (*n* = 1), or both (*n* = 3) were low. Especially low water temperatures below 10–15 °C seem to negatively impact the dolphins’ health, causing an increase in dermal problems (e.g., skin lesions, pox virus, and cold burns), and a decline in calf survival (<14 °C). In addition, cold air temperatures cause respiratory problems, especially in old animals. Sudden changes—a decrease as well as an increase—in water temperature also impact the dolphins’ welfare.

### 2.6. The Dolphin-WET Toolbox

We developed a complete and detailed description of each indicator to facilitate the application for each institution. We refer to this complete set of indicators and their description as well as information on how and when these measurements should be made as the Dolphin-WET Toolbox.

### 2.7. Scoring

In published welfare assessment protocols, aggregation systems and problems of compensation between criteria and indicators have already been highlighted and criticised [49,50]. Given that the final aim of the protocol is to be a routinely applied tool to assess dolphin welfare, we considered a three-level scale to be the best option at finding a balance between repeatability, sensitivity, and accuracy. Of course, an important feature of a good welfare assessment tool is that it must be able to distinguish between different animals in different welfare states. A scoring system with a finer scale would provide more sensitivity and the possibility to distinguish different welfare states. For now, however, some indicators will be scored with a three-level scale, similarly to the Welfare Quality^®^ Assessment Protocols [51,52,53]. However, in some cases, only a two-level scale based on presence or absence can be used, either because presence or absence is the decisive factor and not the degree of impairment (e.g., number of pools), or because there is not enough information to justify a finer scaling (e.g., frequency of synchronous swimming). This approach still allows applicants to easily identify welfare concerns by differentiating between those that must be addressed more urgently and without compromising the feasibility or repeatability of the protocol. However, this does not mean that a finer scale will not be implemented in the future when more data have been collected.

The categories of the three-level scale are defined as follows:

**Score 0. No welfare concern or “adequate”:** the evaluated individual does not show a potential welfare concern in the sub-criteria (or criteria) related to the indicator.

**Score 1. Potential welfare concern or “needs improvement”:** the evaluated individual shows a potential welfare concern in the sub-criteria (or criteria). The needs or requirements related to the criteria seem to not be fully fulfilled, although they are partially addressed and/or addressed with partial success.

**Score 2. Welfare concern or “inadequate”:** the evaluated individual shows a welfare concern in the sub-criteria (or criteria). The needs or requirements related to the criteria are not fulfilled. Addressing actions are absent or ineffective.

For those indicators for which a three-level scale is not applicable, a two-level scale is used instead. A typical example would be an indicator based on the EAAM Standards and Guidelines: if the standards are met, then the score will be 0; if they are not met, then it will be 2.

## 3. Final Tool

The final tool contains 5 principles with a total of 49 indicators (see Appendix A. The Dolphin-WET matrix).

### 3.1. Nutrition

The nutrition principle includes four criteria with a total of six indicators; three of them are animal-based and three are resource-based.

#### 3.1.1. Absence of Prolonged Hunger or Thirst

The nutritional status and its oscillation over the year are important indications of the general condition and, thus, the welfare of the animals.

Body condition scoring (BCS, a visual assessment of muscle and fat cover) and weight are well-accepted welfare measures used for numerous terrestrial and marine animals, both in the wild and under human care [51,52,53,54,55,56]. The Dolphin-WET includes a BCS measure where the assessor visually evaluates the animal during multiple activities (e.g., during training; during free time; and when performing aerial jumps, beaching, and from underwater). A 5-point Likert scale (from emaciated to obese) established by Clegg et al. [35] is used here and translated into a three-level scale, as well as the graphic developed in this study (Credit: Universities Federation for Animal Welfare [UFAW]).

As a second animal-based measure regarding the animals’ nutritional status, the so-called “weight oscillation along the year” indicator assesses how the animal’s weight changes as a percentage of the total weight, using a year’s worth of weight data. Wild dolphins accumulate blubber in the winter as water temperatures decrease [57,58], so some changes in weight throughout the year should be observed, when feeding rations are accorded with water and ambient temperature changes. Given that no studies are available on normal and abnormal weight oscillations in human care settings, a pilot study was conducted in several EAAM facilities to conservatively establish these thresholds. Comparison of the data of healthy animals with those of animals with health problems, and inclusion of husbandry reports and behavioural data, this study revealed that a weight oscillation of 13% or less over a year, and 5% or less across a three-month period, is presumably not a welfare concern, while oscillations outside these thresholds are likely indicative of a welfare issue. A two-level score is applied for this indicator.

#### 3.1.2. Adequate Diet

Dolphins under human care should be fed with fish and cephalopod species that meet their nutritional and hydration needs [59,60], while being palatable. The diet’s nutritional and water content should be monitored and managed when appropriate. An inadequate diet can cause the animal to be lethargic, thirsty, hungry, and/or cause disease and malnutrition. Thus, the indicators for this criterion are whether the dolphin’s diet is calculated using kilocalories based on a two-level score and the blood parameters indicating adequate hydration (e.g., haematocrit and creatinine, see Gulland et al. [61] and Lauderdale et al. [62] for reference values) based on a three-level score.

#### 3.1.3. Adequate Food Quality

Dolphins under human care should be fed with fish that are fit for human consumption, and the quality of purchased batches should be monitored and managed. Fish quality can differ with species, marine location, season, and storage and handling methods. Shelf life is dependent on three criteria: packaging, storage temperature, and fish species [63]. Poor fish quality can cause infection or pathologies in the animal [64,65]. For the fish quality, the selected indicators are microbiological and physicochemical values as required by the EAAM Standards and Guidelines; therefore, a two-level score is used.

#### 3.1.4. Adequate Food Variety

Wild dolphins feed on a large range of prey species, depending on the season, prey availability and quality, and other factors [59]. Feeding a large variety of fish species to dolphins in human care is likely to aid in supplying all necessary nutrients to the animals and to mimic their diet more closely to what they would eat in the wild. A three-level score is used depending on the number of species fed, the adaptation of individual needs, and the provision of food enrichment.

### 3.2. Environment

The environment principle includes seven criteria with a total of nine indicators, seven of which are resource-based and two of which are animal-based.

#### 3.2.1. Safe Environment

An important prerequisite of any animal husbandry is to ensure a safe and secure environment (see EAAM Standards and Guidelines). For example, skin lesions due to injuries caused by sharp objects in the pool and ingestion of foreign bodies are of concern. Here, a two-level score is used.

#### 3.2.2. Adequate Spatial Requirements and Pool Complexity

The presence of adequate space including pool size (horizontally and vertically) and water volume, as well as the possibility to occupy the whole space at any time, are the most important prerequisites to enhance a large range of species-specific behaviours. Large pool dimensions enhance energetic opportunities, like fast swimming, and may decrease aggressive encounters [66]. However, when dolphins are given free choice, they prefer moderate and smaller areas [67]. Even if these results are partly divergent, it is important to note that not only dimensions are important; the complexity of the environment and the division of this area into smaller pools need to be considered [68]. This allows the animals to separate themselves from conspecifics in case of social conflicts. Size, depth, and number of pools are prescribed in the EAAM Standards and Guidelines, so a two-level score is used here.

#### 3.2.3. Social Management

Bottlenose dolphins live in small social units within a fission–fusion society where grouping depends on the age, sex, and the reproductive status of the members (Connor et al., 2000) [69]. Forced isolation has been shown to cause stress in both wild and captive dolphin species [70,71,72]. Accordingly, dolphins should not be kept alone and isolated from the social group for extended periods of time, except, for example, in medical emergencies (see also EAAM Standards and Guidelines). This indicator is evaluated based on records and trainer interviews using a two-level score.

#### 3.2.4. Water Quality

Compliance with correct water parameters is set out in the EAAM Standards and Guidelines. Therefore, a two-level score is applied.

#### 3.2.5. Temperature

Water temperature is an important factor influencing the distribution and seasonal movements of dolphins in the wild [73]. Dolphins seem to tolerate water and air temperature fluctuations without major problems and are found in warm temperate to tropical waters between 10 and 32 °C [74]. However, Yeates and Houser [75] showed that this tolerance is age and sex dependent; mothers with young calves and older animals appear to be more sensitive. This finding was also emphasized by the results of the questionnaires. Furthermore, the surveys revealed that temperatures out of the range proposed by the EAAM, (14–30 °C), and thus also outside the temperature range they would experience in their natural habitat, can negatively affect the behaviour and health of the animals. To take these animal-specific needs into account, a two-level score based on the EAAM Standards and Guidelines seems appropriate and practicable.

#### 3.2.6. Ambient Light

There is growing evidence that lack of shade or light-coloured pools may cause eye damage or exacerbate existing eye lesions in dolphins [76] and other marine animals [77,78,79,80]. The eye and skin are the parts of the body most exposed to ultraviolet (UV) light, and in animals living in open enclosures without significant shade, damage occurs when they are only a few years old [81]. To address these problems and to reduce eye-related issues, less reflective pools and environments, shaded areas, clean water without excessive oxidizers or other irritating byproducts, appropriate feeding methods, and diets with protective antioxidants are recommended [82]. A two-level score is recommended for the provision of sufficient shade and the absence of reflecting colours.

#### 3.2.7. Ambient Noise

**Figure 3:**
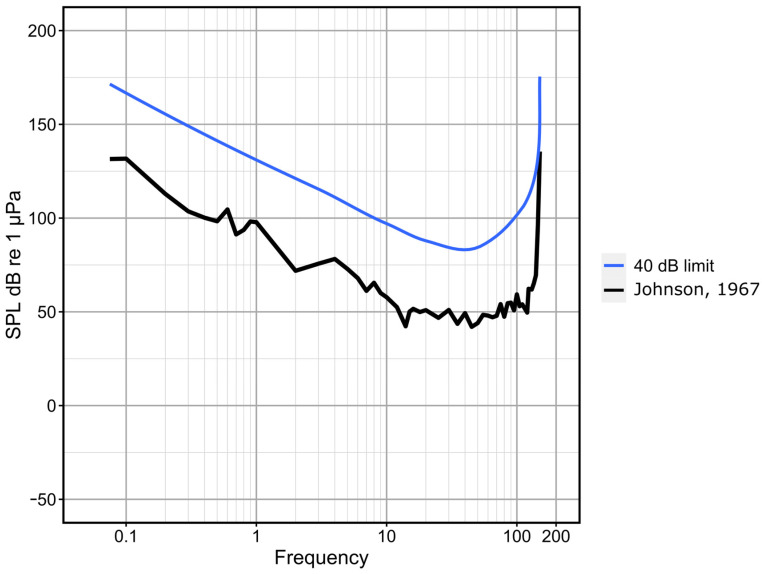
Audiogram of a bottlenose dolphin [90] and the proposed limit of 40 dB above the hearing threshold as described in the “German Expert Opinion on Minimum Requirements for the Keeping of Mammals” (BMEL, 2014).

Given the importance of sound production and processing to the behaviours of dolphins, it is important to create an appropriate acoustic environment when assessing dolphin welfare holistically [83,84]. Therefore, sound measurements play a crucial role in the care of dolphins to determine whether an appropriate acoustic environment is provided. The environment should allow the dolphins to display acoustic behaviours that are important for orientation and communication. While there are some preliminary indications, establishing a scientifically robust definition of the “acoustic comfort zone” for dolphins remains challenging. This goes beyond simply determining the maximum sound pressure level that dolphins can tolerate, it also involves considering the duration and frequency of ambient noises. Only one study measured noise levels in human-managed environments. Houser et al. [85] used a cross-sectional design and focused on determining maximum sound levels in 14 facilities ranging from marine environments to normal dolphinarium pools. The highest recorded sound levels were attributed to whistles and echolocation clicks produced by the animals. Notably, a correlation between group size and noise levels was observed, suggesting that external human-induced noise sources could be ignored as a significant contributor. An important outcome of this study is that it was possible to show that the potential for ambient facility noise to acoustically mask odontocete communication signals and echolocation clicks appears to be low. The study also states that long-term monitoring efforts are essential to understand the variability in noise exposure. It also calls for the investigation of acoustic signals that elicit negative behavioural responses in marine mammals and, thus, affect their welfare. Mooney et al. [86] investigated this question and found that under controlled conditions, mid-frequency sonar can induce temporary hearing loss in a bottlenose dolphin. The effects on hearing were only induced by repeated exposure to intense sonar pulses with total sound exposure levels of 214 dB re: 1 μPa2 s. The exposures also triggered slight behavioural changes. In general terms, it is advisable to measure all these factors by taking regular sound level measurements or implementing an acoustic monitoring system [84,87]. With this approach, changes in the soundscape of the animals that could affect welfare can be assessed more quickly. Despite the ongoing uncertainty surrounding the establishment of upper limits for sound pressure levels, we suggest adopting the values outlined in the “German Expert Opinion on Minimum Requirements for the Keeping of Mammals” [88] as a suitable reference point. According to this document, dolphins should not be kept in environments where the peak sound pressure level at any frequency exceeds 40 dB above their hearing threshold (Figure 3). However, it should be noted that sounds of short duration, lasting only a few seconds, may occasionally exceed this 40 dB limit. This assumption is based on the statements of two experts in marine mammal bioacoustics, who consider the value of 40 dB to be rather conservative and assume that dolphins can tolerate a louder environment (L. von Fersen, personal communication, 14 July 2022). On the other hand, it should be mentioned that mean source levels of bottlenose dolphin whistles have been reported to range from 138 to 158 dB re 1 μPa [89].

Additionally, the duration of sounds, specifically the daily noise exposure level, is a critical factor. This necessitates continuous 24 h recordings to measure anthropogenic noise levels [83,84]. Given the lack of scientifically established standards for the acoustic comfort of dolphins, it is imperative to interpret these values cautiously and to consider them in conjunction with other parameters and behavioural observations. In the absence of new scientific evidence, we adhere to the 40 dB above the hearing threshold as our benchmark for assessing this indicator, employing a two-level score.

It is essential to clarify that we are exclusively addressing underwater noise because we do not deem it relevant to establish tolerable thresholds for airborne sound. Two key reasons support this approach: first, most noise sources to which dolphins are exposed above the water operate in frequency ranges where dolphins have limited hearing ability. Second, dolphins have evolved to excel in underwater hearing, and the layer of air acts as a protective barrier, causing airborne sounds to be significantly attenuated when transmitted underwater [91].

### 3.3. Health

The health principle includes one criterion with a total of 13 indicators, all of which are animal-based. Any health impairment can lead to pain, suffering, or damage and thus to a reduced general condition. These impairments can be short or long term, so both records and a complete veterinary exam of the animal are important in determining the health status.

#### 3.3.1. Correct Locomotion

Normal physiological movement is a sign of a good condition of the musculoskeletal system as well as other organs such as the gastro-intestinal tract and the nervous system. Normal floatability and locomotion are evaluated via underwater and out-of-water observations. Special attention is paid to the movements and mobility of both pectoral flippers, the absence of deformities (e.g., scoliosis), a constant tilt to one side, or external wounds or scars that could affect movement or buoyancy. A two-level score is used, with a score of 0 given if no abnormalities are observed and a score of 2 if there is evidence of them.

Furthermore, records on previous incidents/diseases altering locomotion or floatability during the previous three months provide valuable information. A two-level score is applied by giving a score of 0 if all the movements in the records are within a normal range, and a score of 2 if there are any abnormalities in the records.

#### 3.3.2. Eye Lesions

There are both acute and chronic eye diseases. In both cases, this impairment can lead to reduced vision and can cause pain or be a sign of systemic diseases. For the Dolphin-WET, three indicators are used for the evaluation of eye lesions. First, a two-level score is used during a direct veterinary inspection. A score of 0 is given if no eye lesions are observed, and a score of 2 is given if there is presence of active eye lesions such as opacities, corneal scars, or changes in colour. Second, the bilateral visual ability of the dolphins is checked via testing whether they correctly recognize the visual hand cues given by the trainers. If the response on both eyes is good, then a score of 0 is given. If there is evidence of any abnormalities, then a score of 2 is assigned. Finally, the records on previous eye lesions, incidents, and diseases are reviewed. The two-level score follows the same rules as the other two indicators.

At the time of publication, a doctoral thesis is currently ongoing on the subject of eye health in connection with water quality and other environmental factors.

#### 3.3.3. Mouth Condition (Teeth, Tongue, and Mucosa)

A good mouth condition is related to the teeth, tongue, and mucous membrane. Changes or injuries can lead to painful conditions or a reduced food intake. In addition, symptoms of disease in the oral cavity may be signs of general illness and/or behavioural disorders. The mouth condition is checked through a direct physical exam based on a two-level score. A score of 0 is given if there is no evidence of oral lesions or painful areas in the mouth, and a score of 2 is given if there is evidence of teeth wearing, broken teeth, missing teeth, gingivitis, tongue injuries, fungal lesions, or mucosal lesions, among others. In addition, the records on dental or oral lesions of the previous month are considered and a two-level score is applied as described above.

#### 3.3.4. Gastrointestinal Diseases

An intact gastrointestinal function plays an important role in the animal’s overall health; dysfunction inevitably leads to deterioration in the animal’s welfare. This indicator is assessed through reviewing the records of previous gastric/faecal abnormalities, including the results of gastric cytological evaluations and cultures, and faecal sample cytological evaluations, cultures, and parasitological examinations. A two-level score is used, with a score of 0 assigned if no gastro-intestinal disease is present and score of 2 if there are gastrointestinal diseases.

#### 3.3.5. Respiratory Diseases

Respiratory tract disorders lead to restrictions in the animal and can have various causes, such as bacterial or fungal infections. This criterion is assessed by using three indicators: a direct physical examination (i.e., visual blowhole evaluation and auscultation), the forced expiration test, and a review of the records on previous respiratory issues. For each indicator, a two-level score is used: A score of 0 is assigned in the case of absence and 2 is assigned if there are signs of respiratory disease.

#### 3.3.6. Generic/Systemic/Other Diseases

To exclude other systemic diseases, it is necessary to examine the skin and all body openings and to evaluate the results of the most recent haematology. Hence, the first indicator of this criterion is a direct physical exam of the skin and natural openings, including the global skin condition (e.g., colour, number and severity of viral lesions, major wounds, cracking, relative desquamation, scars, thermal ischemic necrosis, etc.), genital slit (e.g., exudates, mucosal colour, and mucosal lesions), ears (e.g., exudates, pain to touch, and bulging), and rostrum (e.g., wounds and callosity). It is important to evaluate not only the colour and integrity of the skin and mucosa, but fresh lesions and recent scars, hematomas, relative desquamation, and indications of viral diseases that appeared during the last three months since the previous evaluation. As in all the other health indicators, a two-level score is used, with a score of 0 assigned in the absence of all these lesions and a score of 2 assigned if any indications of a disease are present.

For the second indicator, the records on previous incidents during the last three months are reviewed and the same scores are assigned in the case of the absence or presence of such diseases. These records should always include a complete blood profile containing a complete panel on haematology, biochemistry, and coagulation parameters (30 in total), including the most relevant markers or inflammation (see Gulland et al. [61]). All parameters measured should remain in the normal range for the species as stated by Gulland et al. ([61]; Appendix 1, pp. 1003–1005) and, if available, within the individual reference values/ranges according to previous analysis (at least including a 1 year period and ideally more) under the same lab and technique.

### 3.4. Behaviour

As mentioned previously, we selected 15 indicators (1 resource-based indicator and 14 animal-based indicators) to assess the behaviour of bottlenose dolphins (Table 1). Furthermore, we agreed that this principle evaluates whether species-specific behaviours, such as affiliative behaviour and play behaviour, are displayed, or encouraged. At the same time, the occurrence of abnormal behaviours that might indicate poor welfare are also assessed, including stereotypic behaviour and intense and repetitive aggression.

Most of the presented indicators must be assessed and evaluated through behavioural observations using a standardized protocol for behavioural data collection that includes clear definitions and descriptions of each behaviour and specific recording criteria. The protocol must allow for the assessment of presence/absence as well as frequency of the sampled behaviour. The observers need to be trained to recognize and interpret dolphin behaviours in order to ensure a high inter-observer reliability and to minimize observer bias. Also, ensuring that the dolphins’ behaviour is not affected by the presence of the observer is essential. Therefore, the dolphins need to be habituated to the presence of the observer in case it is a familiar person, such as a trainer, researcher, or veterinarian, who, unlike usually, does not interact with the animals.

Another challenge was to define the thresholds to score the observed behaviours on a scale from poor to high welfare (using frequencies, duration, etc.). Some studies [33,92] suggest that a significant increase or decrease in certain behaviours (e.g., a decrease in affiliative behaviour, see Huettner et al. [18]) can be an indication of a welfare problem. Unfortunately, although there are many studies that have examined the behaviour of dolphins in human care, and which behaviours are displayed, little is known about how often these behaviours (e.g., social play, flipper rubbing, or socio-sexual interactions) should or should not occur. Lauderdale et al. [93] generated the first comprehensive reference values and activity budgets for bottlenose dolphins living under human care. Still, based on our limited knowledge on the expected behavioural spectrum and the general occurrence, frequency, or duration of certain appropriate behaviours of dolphins under human care and their significance for animal welfare, our protocol currently only focuses on the presence of behaviours that reflect a positive welfare state and the absence of behaviours that reflect a negative welfare state during the behavioural observations, assessed with a two-level score.

#### 3.4.1. Display of Important Behaviours That Reflect a Positive Welfare State

##### Exploratory Behaviours

Exploratory behaviour describes the actions that an animal performs to obtain information about a new object, environment, or individual through using its different senses of perception [94]. Under human care, environmental enrichment promotes exploratory behaviour in bottlenose dolphins [95], with some interindividual variation related to their personality [96,97], the type of introduced objects [16], and the sex and/or age of the individuals [17]. Furthermore, environmental enrichment can increase behavioural diversity and provide animals with opportunities to make choices and to have some control over their environment (see Lauderdale et al. [98]). Based on the EAAM Standards and Guidelines, this indicator is awarded a score of 0 if the facility has an enrichment programme in place and the dolphins display exploratory behaviour during enrichment programme sessions. If the dolphins do not display exploratory behaviour, a score of 1 is given. Finally, a score of 2 is assigned if the facility has no environmental enrichment programme.

##### Affiliative Behaviours

Affiliative behaviours in dolphins include synchronized pair/group swimming with or without flipper rubbing [99,100,101,102,103,104], contact swimming [105], and social play [106]. Some affiliative behaviours such as slow synchronized swimming [33,107,108] as well as the general frequency of social behaviours in general (see Huettner et al. [18]) have been validated as reliable positive welfare indicators in dolphins. Thus, when applying the Dolphin-WET, whether the dolphins display affiliative behaviours (score of 0) or not (score of 2) is evaluated via behavioural observations.

##### Play Behaviours

In many species, play is mainly displayed by juvenile animals, but dolphins display playful behaviours across all age classes and in both sexes [109]. While older individuals develop more complex play behaviours [97], play can involve solitary (locomotory) play, observational play, object play, and social play [106,110,111]. As play most likely occurs when an animal does not experience ultimate threats (e.g., predation and hunger), the occurrence of play behaviour is considered an indicator of a positive welfare state [92,112,113]. In dolphins, social and object play are negatively correlated with different stressors [92,114,115], highlighting their importance as a positive welfare indicator. Thus, during the evaluation, each facility should assess whether the dolphins demonstrate play behaviour (score of 0) or do not (score of 2).

##### Socio-Sexual Behaviours

Like affiliative behaviour, sexual behaviour in dolphin societies has been suggested to maintain social dynamics, bonding, and group cohesion ([116,117]; for a review see, Manitzas Hill et al. [118]). Socio-sexual behaviour is defined through opposite and same-sex genital contact and includes mounting, goosing, push-ups, petting, and rubbing of the genital area (see Harvey et al. [119] and Mann [117]). Again, the scoring is based on the presence or absence of this behaviour, with the presence indicating an adequate welfare state (score of 0) and the absence indicating an inadequate welfare state (score of 2).

##### Maternal Behaviour

Bottlenose dolphin calves associate strongly with their mother during the first years and are usually nursed for 3–6 years [120]. The relationship between a mother and her calf is complex and variable and depends on the mother’s experience (primiparous vs. multiparous) and the calf’s sex [121]. Young calves mainly swim in the echelon position (calf parallel to the mother, head next to the mother’s dorsal fin, and close to the mother) [120,122] until about halfway through their first year. With increasing age and size, the time spent in the echelon position usually decreases and the calf favours the so-called infant position (or mother–calf position, [120,121,122]).

If a mother–calf dyad is present, then behavioural observations should be used to assess if the mother is displaying maternal behaviours (e.g., close proximity swimming, nursing behaviour, and affiliative interactions between mother and calf). If maternal behaviour is observed, the dolphin receives a score of 0; if there are no maternal behavioural patterns, then the score is 2.

#### 3.4.2. Absence of Abnormal Behaviours/Behaviours Indicative of Poor Welfare

To assess negative welfare indicators such as stereotypies (i.e., abnormal repetitive behaviour), regurgitation, and intense and repetitive aggressive interactions, we decided to score their presence (i.e., a score of 2) or absence (i.e., a score of 0).

##### Stereotypical and Abnormal Repetitive Behaviours

Stereotypies or abnormal repetitive behaviours remain poorly studied in dolphins or cetaceans. Nevertheless, in a wide range of other species, these behaviours have been extensively linked to other factors (i.e., personality and anticipatory behaviour [123]) and ultimately to poor welfare [124]. In addition, repetitive food regurgitation may also have a negative effect on gastrointestinal health because it could cause oesophageal ulcers or dental problems [125]. Thus, whether a dolphin displays any form of stereotypical or other abnormal behaviours (a score of 2) or does not (a score of 0) needs to be evaluated.

##### Absence of Receiving/Displaying Aggressive Behaviours Causing Negative Consequences

Agonistic or aggressive behaviours are part of social animals’ interactions [69]. Although aggression is generally considered to be low among dolphins [119,126,127], aggression can cause negative effects due to its chronicity or due to a high intensity (e.g., inappetence, severe injuries, or social isolation of the receiver), and can result from underlying social problems that may be connected to other stressors, also indicating poor welfare [72]. While for the receiver, the connection of receiving aggression causing negative consequences with the welfare is more obvious, for the aggressor it may not be so clear how the animal’s welfare is affected. There is a welfare issue for the animal initiating aggressive behaviour if it is also injured. If not, we can only assume that its welfare is compromised in some ways that compel it to behave aggressively at high frequencies.

Tooth (rake) marks, which are typically characterised by linear or parallel scratches on the skin of dolphins, are among the most common injuries resulting from social aggression observed in wild dolphins and animals under human care [35,126,128,129]. While they are considered a common aspect of social behaviour in dolphins under human care and in the wild, they are an established indicator to measure aggression received by conspecifics because severe rake marks can lead to injuries, infections, or other health issues in dolphins [35,126,128]. Thus, we recommend a three-level score based on Clegg et al. [35] to quantify the severity and frequency of the scarring of fresh tooth rakes on a dolphin during the Dolphin-WET evaluation.

##### Social Isolation

Dolphins are highly social animals known for their complex social structures. When dolphins avoid areas occupied by other individuals, it may indicate the presence of social stress or aggression within the group. Social conflicts can lead to injuries, increased stress levels, and reduced overall welfare [69]. For the Dolphin-WET, signs of social isolation during the last three months are recorded through interviews with the trainers. If incidents were observed, then a score of 2 is given; if not, a score of 0 is given.

##### Avoidance of Certain Pool Areas

Other than avoiding certain pool areas due to the presence of other individuals, the intentional avoidance of a specific area of the pool might also indicate a welfare concern. Factors such as visitor presence [130], visitor interaction activities [131,132], and underwater noise [133] may lead to avoidance behaviour. For the evaluation, we propose a two-level score based on trainer interviews. A score of 0 is awarded if the evaluated dolphin generally uses all available pool regions. If a dolphin actively avoids a certain area, then a score of 2 is assigned.

### 3.5. Mental State

As described by Mellor et al. [5], several factors from each of the other four domains have specific negative or positive effects on the animal’s mental state. Additional indicators are needed to assess the mental state. For the Dolphin-WET, we chose four criteria with six indicators (one resource-based indicator and five animal-based indicators) that may indicate a negative or positive affective state in bottlenose dolphins.

#### 3.5.1. Positive Human–Animal Relationship

In zoos, human–animal interactions between keepers and the animals under their care occur in a variety of different settings. Dolphins engage in human–animal interactions more frequently than other zoo animals. The nature and quality of these relationships can have an important impact on animal welfare [134]. In dolphins, Clegg et al. [135] and Miller, Lauderdale, Mellen et al. [24] have already shown that daily interactions during training sessions and other activities can have a positive effect on the behaviour and welfare of the animals. Thus, measuring the quality of human–animal interactions should be included in a holistic animal welfare evaluation tool because it can provide valuable insights into the animal’s welfare state [5]. The parameter “willingness to participate” (WtP) has already been validated as an effective welfare indicator [31] because it predicts early changes in a dolphin’s health [15] and is linked to six alerting factors [30]. WtP is generally assessed on a 5-point Likert scale and describes the dolphin’s motivation and enthusiasm during training sessions; it ranges from 0 when a dolphin was not present during the entire training to 4 when a dolphin performed all asked behaviours with high motivation throughout the session [15]. Based on existing data, we defined a two-level score: the score is 0 if the average WtP across 5 days is ≥2.5 or 2 if the WtP is <2.5.

#### 3.5.2. Behaviours Linked to Positive Mental States

Occurrences of slow synchronous swimming [107] and gentle rubbing behaviours [107,136,137,138] have been linked to positive affective states. Like the indicators for the principles of behaviour, a two-level score is used here. A score of 0 is given if the behaviours are present during behavioural observations, and a score of 2 is given if the behaviours are absent.

Anticipatory behaviours have also been linked to cognitive biases and can be easily induced and measured [139,140]. Through using “The Fast Technique” developed by Bigiani and Pilenga [139], one can observe whether a dolphin displays anticipatory behaviours when enrichment objects are presented before the dolphin can interact with them. If the animal exhibits anticipatory behaviours, a score of 0 is given; if not, a score of 2 is given.

#### 3.5.3. Swimming Behaviours Linked to Negative Mental States

Swimming features [18,33] such as fast swimming [18] or repetitive tail slapping [141,142] could also indicate the negative emotional state of the dolphins. So, through conducting behavioural observations and by scoring the presence (score of 2) or absence (score of 0) of these behaviours, we can infer negative or positive affective states in bottlenose dolphins [143].

#### 3.5.4. Management Policy towards Choice and Control

Lastly, the facility’s policy towards choice and control is evaluated. Recent advances in zoo animal research have demonstrated that affording animals the opportunities to choose between two or more possibilities—for example, staying in the inside or outside part of their enclosure—benefits their overall welfare [144,145,146]. To grant dolphins a degree of choice and control and to respond to individual preferences, they could, for example, be allowed to choose between different enrichment devices or types of positive reinforcement. Granting choice and control could also mean letting a dolphin decide whether it participates in a certain type of training activity [147,148]. This resource-based indicator is evaluated using a two-level score: a score of 0 indicates that the dolphins have the possibility of environmental choice and control, while a score of 2 indicates that they do not.

#### 3.5.5. Qualitative Behaviour Assessment (QBA)

Another possible indicator for measuring the emotional state of animals is QBA. Following a “whole-animal” approach, QBA evaluates the demeanour of an animal as a response to the animal’s environment [149,150]. QBA has already been used to assess the emotional state in various zoo species welfare assessments, including for elephants [2], polar bears [151], and giraffes [152]. Rose and Riley [153] suggested that QBA could play a useful role in evaluating the effect of environmental design and enrichment on zoo animal welfare. In general, QBA scoring is based on a list of species-relevant terms related to the different dimensions of emotion. The term list can either be generated ad hoc by individual observers based on observations (free choice profiling, see Clarke et al. [154]), or as a fixed-term list that may be constructed by expert or stakeholder consultation and/or by scanning the relevant literature [155,156].

At this time, QBA is not yet part of the Dolphin-WET, but we created a list of 17 terms (e.g., “apathetic”, “playful”, “excited”, and “sad”) by reviewing the literature and by including a pilot study on bottlenose dolphins [157] and experts’ assessments. In an ongoing study, we will evaluate these terms alongside more established behavioural and physiological indicators so that we can include QBA as a welfare indicator in the Dolphin-WET.

## 4. Creation of an App

Members of the Dolphin-WET group based at Nuremberg Zoo, Germany, have been collaborating with the University of Erlangen for many years, including the Machine Learning and Data Analytics Lab (MaD Lab, see Zuerl et al. [158,159]). In 2023, members of the Innovation Lab, a student course offered by the MaD Lab, in which students learn to develop practical systems and software engineering problems, agreed to support the implementation of the Dolphin-WET by creating a mobile application for the use of the tool. Through streamlining the process of data collection and analysis, the app decreases time and personnel needs and enables comprehensive insights into animal welfare states in real time. As a result, the app accelerates the overview of individual welfare states and allows for swift intervention and decision-making, ultimately leading to more effective and timely improvements in animal welfare practices. The development started in April 2023 and resulted in a first prototype in August 2023. First, it had to be clarified which functions the app should cover. While developing the app, it was necessary to address what the app functionalities should cover. The EAAM Welfare Committee and the Innovation Lab defined three main functionalities: (1) easy, intuitive, and concise documentation of the Dolphin-WET results; (2) comprehensive visualisation and overview of the welfare data for each individual; and (3) guidance for first time Dolphin-WET users through the testing and documentation process using easy-to-understand instructions and examples (the Dolphin-WET Toolbox and scoring system). A simplistic and intuitive approach was followed to achieve concise documentation. The application contains relevant information for immediate on-site application. Furthermore, the Innovation Lab team precisely followed the procedures of the Dolphin-WET to adapt the app towards the test workflow. Following these two approaches, the app contains just three tabs: “Documenting Data”, “Visualizing Data”, and “Dolphin Data”.

## 5. Conclusions

The Dolphin-WET is a species-specific welfare assessment tool created to evaluate the welfare of bottlenose dolphins under human care. In line with the increasing importance of making animal welfare more tangible, the Dolphin-WET represents a holistic tool that allows a user to conduct a welfare assessment for dolphins under human care easily, regularly, and without the need of external assistance.

The Dolphin-WET consists of 49 indicators covering the Five Domains Model (i.e., nutrition, environment, health, behaviour, and mental state). Most of these indicators are animal-based (37); the remaining 12 indicators are resource-based. Although some indicators still await scientific validation, one of the main advantages of the tool is that it allows a user to identify and address specific welfare issues for each individual.

The Dolphin-WET is intended for regular applications by animal care experts, including zookeepers, biologists, ethologists, and veterinarians, to ensure the consistent and ongoing monitoring of a dolphin’s welfare as part of the in-house welfare assessments. Through combining record-based data (e.g., veterinary records and trainer reports) with real-time information (e.g., on-site evaluation of health status and behavioural observations), a comprehensive and detailed view of an individual dolphin’s welfare state since the last evaluation can be assessed, enhancing the effectiveness and sensitivity of the assessment process. This approach allows a user to track the effects of implemented interventions aimed to improve the welfare of each animal and to address their specific needs and problems.

We must note that the Dolphin-WET is designed to be a living tool that will continue to be refined and improved, incorporating new scientific knowledge on dolphin welfare to increase the sensitivity of the indicators. Furthermore, there is the possibility of incorporating new welfare indicators, such as QBA, into the tool. Regular evaluations and adaptations of the protocol are expected to provide even more reliable and accurate information on the welfare status of bottlenose dolphins. At the same time, the tool can be used as the basis of and adapted for the holistic welfare evaluations of other zoo species.

## Figures and Tables

**Table 1:** Overview of the number of resource-based and individual-based welfare indicators used in the final list of indicators.

Principle	Indicators	Resource-Based	Animal-Based
Nutrition	6	3	3
Environment	9	7	2
Health	13	0	13
Behaviour	15	1	14
Mental state	7	1	6
**Total**	**50**	**12**	**38**

*^1^ Based on the German “Gutachten über die Mindestanforderungen an die Haltung von Säugetieren” (BMEL, 2014).

*^2^ Including complete haematology, biochemistry, erythrocyte sedimentation rate, and fibrinogen determinations, as well as inflammatory markers (total white blood cell count, white blood cell differential count, band neutrophils (%), reticulocytes (%), haemoglobin, alkaline phosphatase, albumin, fibrinogen, and iron), biological data, or complementary diagnostic techniques (ultrasound, radiography, endoscopy, thermography, computed tomography scan, microbiology, hormonal analysis, molecular techniques, etc.).

^(^*^3)^ Displacement behaviour includes self-directed behaviours displayed when an animal has a conflict between two motivations—for example, the desire to approach an object while at the same time being fearful of that object.

## Data Availability

Not applicable since this paper is mainly a theoretical paper.

## Appendix A. The Dolphin-WET Matrix

**Nutrition**			
**Absence of prolonged hunger or thirst**			
*Correct amount of food eaten*			
Indicator	Validation	Scoring description	Score
Body condition score (BCS)	Peer review	BCS of 3 = adequate	0
		BCS of 2 (underweight) or 4 (overweight)	1
		BCS of 1 (emaciated) or 5 (obese)	2
Weight oscillation throughout the year	Management-based expertise	Body weight oscillation (BWOS): ≤13% throughout the year or ≤5% in a 3-month period	0
		BWOS: >13% throughout the year or >5% in a 3-month period	2
**Adequate diet**			
*All water and nutritional requirements are covered*			
Indicator	Validation	Scoring description	Score
Kilocalories	Standards and Guidelines	Diet designed based on the EAAM Standards and Guidelines	0
		Diet not designed based on the EAAM Standards and Guidelines	2
Blood parameters for adequate hydration	Peer review	Within the range	0
		10% out of range	1
		>10% out of range	2
**Adequate food quality**			
*Analysis of fish and shelf life*			
Indicator	Validation	Scoring description	Score
Food quality, microbiology, physicochemical analysis	Standards and Guidelines	Fulfilled according to the EAAM Standards and Guidelines	0
		Not fulfilled according to the EAAM Standards and Guidelines	2
**Adequate food variety**			
*Adequate alimentary variability*			
Indicator	Validation	Scoring description	Score
Food variety throughout the year	Management-based expertise	At least five species are fed throughout the year, each individual’s diet is adapted to its nutritional needs and preferences, the amount of food fed varies between sessions, and part of the diet is given via enrichment	0
		At least three, but no more than five, species are fed throughout the year, each individual’s diet is adapted to its nutritional needs, but the amount of food provided to the dolphins during each session is the same, and favourite species/animal preferences are neglected	1
		Only three or less species of food are fed throughout the year, no variation throughout the year, and diet is not adapted to individual preferences/specific need	2
**Environment**			
**Safe environment**			
*Health-related safe environmental design*			
Indicator	Validation	Scoring description	Score
Enclosure and barrier safety and maintenance	Standards and Guidelines	All criteria are met according to the EAAM Standards and Guidelines (see Section 5)	0
		At least one criterion is not met according to the EAAM Standards and Guidelines (see Section 5)	2
No foreign body ingestion		Absence in records	0
		Presence in records (last 3 months)	2
**Adequate spatial requirements and pool complexity**			
*Possibility to freely move between areas, resting place, and engaging environment*			
*Possibility to perform desired behaviours, including fast swimming, jumping, social behaviours, and synchronised behaviour*			
*Possibility of escaping from conspecifics*			
Indicator	Validation	Scoring description	Score
Pool dimension, pool design, number of pools, available access to pools, and group management	Standards and Guidelines	All criteria are met according to the EAAM Standards and Guidelines (see Section 5)	0
		At least one criterion is not met according to the EAAM Standards and Guidelines (see Section 5)	2
**Social management**			
*Evidence of forced loneliness*			
Indicator	Validation	Scoring description	Score
Absence of forced loneliness based on records/ trainer interviews		Dolphin was not separated or only separated during medical emergencies or for research purposes for short periods of time (<1 h/day) during the last 3 months	0
		Dolphin was separated for longer periods of time (>1 h/day) during the last 3 months	2
**Water quality**			
*Adequate water pH*			
*Adequate water oxidation reduction potential (ORP) levels*			
*Absence of toxic water levels of total chlorine, free chlorine, and combined chlorine*			
*Absence of abnormal water levels of coliforms*			
*Absence of abnormal water levels of Escherichia coli*			
*Adequate salinity*			
Indicator	Validation	Scoring description	Score
Water quality parameters	Standards and Guidelines	All criteria are met according to Section 9 of the EAAM Standards and Guidelines	0
		At least one criterion is not met according to Section 9 of the EAAM Standards and Guidelines	2
**Temperature**			
*Adequate water temperature*			
Indicator	Validation	Scoring description	Score
Water temperature levels	Standards and Guidelines	All criteria are met according to Section 9 of the EAAM Standards and Guidelines	0
		At least one criterion is not met according Section 9 of to the EAAM Standards and Guidelines	2
**Ambient light**			
*Presence of/access to shaded areas*			
Indicator	Validation	Scoring description	Score
Sufficient shade provided and accessible in case it is needed	Standards and Guidelines	All criteria are met according to Section 5 of the EAAM Standards and Guidelines	0
		At least one criterion is not met according to Section 5 of the EAAM Standards and Guidelines	2
*Absence of light-reflecting colours for pool walls*			
Indicator	Validation	Scoring description	Score
Absence of reflecting colours	Standards and Guidelines	All criteria are met according to Section 5 of the EAAM Standards and Guidelines	0
		At least one criterion is not met according Section 5 of to the EAAM Standards and Guidelines	2
**Ambient noise**			
*Adequate degree of underwater noise*			
Indicator	Validation	Scoring description	Score
40dB above hearing threshold *^1^	Management-based experience	No noise above the 40 dB hearing threshold for more than 1 min/day	0
		Noise above the 40 dB hearing threshold for more than 1 min/day	2
*^1^ Based on the German “Gutachten über die Mindestanforderungen an die Haltung von Säugetieren” (BMEL, 2014).			

**Health**			
**Absence of diseases**			
*Correct locomotion*			
Indicator	Validation	Scoring description	Score
Normal floatability and displacement movement test	Peer review	Verification of normal movements	0
		Evidence of any locomotive/floating abnormalities	2
Records on previous incidents/diseases altering locomotion or floatability	Peer review	Absence of any locomotive/floating abnormalities in records	0
		Evidence of any locomotive/floating abnormalities in records	2
*Eye lesions*			
Indicator	Validation	Scoring description	Score
Direct veterinary inspection	Peer review	Absence of active eye lesions	0
		Presence of active eye lesions	2
Normal responses in both eyes to visual cues	Peer review	Normal response	0
		Evidence of any abnormalities	2
Records on previous eye lesions/incidents/diseases	Peer review	Absence of previous incidents in records during the last 3 months	0
		Evidence of previous incidents in the records during the last 3 months	2
*Mouth condition (teeth, tongue, and mucosa)*			
Indicator	Validation	Scoring description	Score
Direct physical exam of mouth	Peer review	Absence of oral lesions or painful areas in the mouth (including teeth) based on direct physical exam	0
		Presence of oral lesions or painful areas in the mouth (including teeth) based on direct physical exam	2
Records on previous dental or oral lesions	Peer review	Absence of previous incidents in the records during the last 3 months	0
		Evidence of previous incidents in the records during the last 3 months	2
*Gastrointestinal tract diseases*			
Indicator	Validation	Scoring description	Score
Records of previous gastric/faecal abnormalities	Peer review	Absence of previous incidents in the records during the last 3 months	0
		Evidence of previous incidents in the records during the last 3 months	2
*Respiratory diseases*			
Indicator	Validation	Scoring description	Score
Direct physical exam including blowhole visual evaluation and respiration performance	Peer review	Absence of any signs or evidence of respiratory disease during direct physical exam	0
		Presence or signs of respiratory disease during direct physical exam	2
Forced expiration test	Peer review	Absence of abnormalities	0
		Evidence of any abnormalities	2
Records on previous respiratory lesions/incidents/diseases	Peer review	Absence of previous incidents in the records during the last 3 months	0
		Evidence of previous incidents in the records during the last 3 months	2
*Generic/systemic/other diseases*			
Indicator	Validation	Scoring description	Score
Direct physical exam	Peer review	Absence of external active signs of disease on skin or other natural openings on direct physical exam including global skin condition (colour, number and severity of viral lesions, major wounds, cracking, relative desquamation, scars, thermal ischemic necrosis, etc., excluding rake marks), genital slit (exudates, mucosal colour, and mucosal lesions), ears (exudates, pain to touch, and bulging), and rostrum (wounds and callosity)	0
		Presence of external active signs of disease on skin or other natural openings on direct physical exam including global skin condition (colour, number and severity of viral lesions, major wounds, cracking, relative desquamation, scars, thermal ischemic necrosis, etc., excluding rake marks), genital slit (exudates, mucosal colour, and mucosal lesions), ears (exudates, pain to touch, and bulging), and rostrum (wounds and callosity)	2
Records (complete blood work *^2^)	Peer review	Absence of previous signs/diseases in the records during the last 3 months	0
		Evidence of previous signs/diseases in the records during the last 3 months	2
*^2^ Including complete haematology, biochemistry, erythrocyte sedimentation rate, and fibrinogen determinations, as well as inflammatory markers (total white blood cell count, white blood cell differential count, band neutrophils (%), reticulocytes (%), haemoglobin, alkaline phosphatase, albumin, fibrinogen, and iron), biological data, or complementary diagnostic techniques (ultrasound, radiography, endoscopy, thermography, computed tomography scan, microbiology, hormonal analysis, molecular techniques, etc.).			

**Behaviour**			
**Display of important behaviours that reflect a positive welfare state**			
*Display of exploratory behaviour*			
Indicator	Validation	Scoring description	Score
Adequate environmental enrichment programme inducing exploratory behaviour	Peer review	Presence of an enrichment programme according to the EAAM Standards and Guidelines and display of exploratory behaviours	0
		Presence of an enrichment programme according to the EAAM Standards and Guidelines, but no display of exploratory behaviour	1
		No enrichment programme	2
*Display of affiliative behaviour, play, or socio-sexual behaviour*			
Indicator	Validation	Scoring description	Score
Affiliative behaviour	Peer review	Dolphin displays affiliative behaviours (e.g., pair swimming, flipper rubbing, etc.) during observations	0
		Dolphin does not display affiliative behaviours (e.g., pair swimming, flipper rubbing, etc.) during observations	2
Play behaviour	Peer review	Dolphin displays play behaviour (social play, bubble ring play, object play, etc.) during observations	0
		Dolphin does not display play behaviour (social play, bubble ring play, object play, etc.) during observations	2
Socio-sexual behaviour	Peer review	Dolphin displays socio-sexual interactions (petting, goosing, etc.) during observations	0
		Dolphin does not display socio-sexual interactions (petting, goosing, etc.) during observations	2
*Display of maternal behaviour when justified*			
Maternal behaviour	Peer review	Display of appropriate maternal behaviour towards the calf (echelon swimming, nursing, etc.)	0
		Absence of appropriate maternal behaviour towards the calf	2
**Absence of abnormal behaviours/behaviours indicative of poor welfare**			
*Absence of repetitive abnormal behaviours*			
Indicator	Validation	Scoring description	Score
Displacement behaviour ^(^*^3)^	Peer review	Dolphin does not display this behaviour during the last 3 months based on trainer interviews	0
		Dolphin displays the behaviour	2
Oral stereotypic behaviour	Peer review	Dolphin does not display oral stereotypic behaviour (e.g., chewing on gates, hoses, and toys)	0
		Dolphin displays oral stereotypic behaviour	2
Repetitive body movement	Peer review	Dolphin does not display this behaviour (e.g., circling)	0
		Dolphin displays the behaviour	2
Frequent, repetitive, and intense self-grooming behaviour	Peer review	Dolphin does not display frequent, repetitive, and intense self-grooming	0
		Dolphin displays frequent, repetitive, and intense self-grooming (e.g., excessive bottom rubbing)	2
Regurgitation/Reingestion	Peer review	Dolphin does not regurgitate	0
		Dolphin regurgitates	2
*Absence of receiving aggressive behaviours, causing negative consequences*			
Indicator	Validation	Scoring description	Score
Receiving aggressive behaviour		Dolphin does not receive aggressive behaviour (e.g., biting, hitting, and slapping) during observations	0
		Dolphin receives aggressive behaviour (e.g., biting, hitting, and slapping) during observations	2
Rake marks (social-related marks)	Peer review	0%–15% new rake marks and < 30% old wounds	0
		15%–20% new rake marks and > 30% old wounds	1
		> 20% new rake marks	2
			
*Absence of displaying aggressive behaviours, causing negative consequences*			
Indicator	Validation	Scoring description	Score
Displaying aggressive behaviour	Peer review	Dolphin does not display aggressive behaviour (e.g., biting, hitting, and slapping) during observations	0
		Dolphin displays frequent, repetitive, and intense aggressive behaviour (e.g., biting, hitting, and slapping) during observations	2
*Evidence of social isolation*			
Indicator	Validation	Scoring description	Score
Social isolation		Absence of social isolation during the last 3 months based on trainer interviews	0
		Presence of other animals lead to avoidance of certain pool areas during the last 3 months based on trainer interviews	2
*Evidence of avoidance of certain pool regions*			
Indicator	Validation	Scoring description	Score
Avoidance of pool areas		Absence of active avoidance of certain pool areas during the last three months	0
		Dolphin **avoids** certain pool areas on a consistent basis	2
^(^*^3)^ Displacement behaviour includes self-directed behaviours displayed when an animal has a conflict between two motivations—for example, the desire to approach an object while at the same time being fearful of that object.			

**Mental state**			
**Positive human–animal relationship**			
*Positive animal–trainer relationship*			
*Positive animal–visitor relationship*			
Indicator	Validation	Scoring description	Score
Willingness to participate (WtP)	Peer review	Average WtP over 5 days (same days as behavioural observations) is ≥2.5	0
		Average WtP over 5 days (same days as behavioural observations) is <2.5	2
**Behaviours linked to positive affective states**			
*Evidence of behaviours indicating positive mental state*			
Indicator	Validation	Scoring description	Score
Slow synchronous swimming, slow contact swimming, slow circular swimming	Peer review	Dolphin displays slow synchronous swimming, slow contact swimming, and slow circular swimming during observations	0
		Dolphin does not display slow synchronous swimming, slow contact swimming, and slow circular swimming during observations	2
Gentle rubbing behaviour	Peer review	Dolphin displays gentle rubbing behaviours during observations	0
		Dolphin does not display gentle rubbing behaviours during observations	2
Anticipatory behaviour	Peer review	Dolphin displays anticipatory behaviour during observations	0
		Dolphin does not display anticipatory behaviour or displays intense and long-lasting anticipatory behaviour during observations	2
**Swimming behaviours linked to negative affective states**			
*Evidence of behaviours indicating negative mental state*			
Indicator	Validation	Scoring description	Score
High frequency of fast swimming behaviour	Peer review	Dolphin does not exhibit high frequencies of fast swimming behaviour during observations	0
		Dolphin exhibits high frequencies of fast swimming behaviour during observations	2
Intense and repetitive tail slapping behaviour	Peer review	Dolphin does not display intense and repetitive tail slapping behaviour during observations	0
		Dolphin displays intense and repetitive tail slapping behaviour during observations	2
**Abilities of environmental choice and control**			
*Environmental choice and control*			
Indicator	Validation	Scoring description	Score
Evaluation of choice and control based on trainer survey	Peer review	Facility promotes policies or management favouring choice and control, not forcing the animal towards any type of participation in any regular activity except for medical purposes as needed.Choice is promoted in other daily care activities such as feeding, enrichment, or companionship	0
		Facility does not promote policies or management favouring choice and control, not forcing the animal towards any type of participation in any regular activity except for medical purposes as needed. Choice is not promoted in other daily care activities such as feeding, enrichment, or companionship.	2
